# Effects of maternal and infant co-infections, and of maternal immunisation, on the infant response to BCG and tetanus immunisation

**DOI:** 10.1016/j.vaccine.2010.10.047

**Published:** 2010-12-16

**Authors:** Alison M. Elliott, Patrice A. Mawa, Emily L. Webb, Margaret Nampijja, Nancy Lyadda, Joseph Bukusuba, Moses Kizza, Proscovia B. Namujju, Juliet Nabulime, Juliet Ndibazza, Moses Muwanga, James A.G. Whitworth

**Affiliations:** a MRC/UVRI Uganda Research Unit on AIDS, P.O. Box 49, Entebbe, Uganda; b London School of Hygiene & Tropical Medicine, Keppel Street, London WC1E 7HT, UK; c Entebbe Hospital, P.O. Box 29, Entebbe, Uganda

**Keywords:** BCG, Tetanus, Immunisation

## Abstract

Some vaccines show poor efficacy in tropical countries. Within a birth cohort in Uganda, we investigated factors that might influence responses to BCG and tetanus immunisation. Whole blood assay responses to crude culture filtrate proteins of *Mycobacterium tuberculosis* (cCFP)) and tetanus toxoid (TT) were examined among 1506 and 1433 one-year-olds, respectively. Maternal *Mansonella perstans* infection was associated with higher interleukin (IL)-10 responses to both immunogens but no reduction in gamma interferon (IFN-γ), IL-5 and IL-13 responses; other maternal helminth infections showed little effect. Tetanus immunisation during pregnancy was associated with higher infant responses to TT; maternal BCG scar (from past immunisation) with lower infant IL-5 and IL-13 responses to cCFP. IFN-γ, IL-5 and IL-13 to TT were reduced in HIV-exposed-uninfected infants; infant malaria and HIV were associated with lower IFN-γ, IL-5 and IL-13 responses to both immunogens. We conclude that maternal helminth infections are unlikely to explain poor vaccine efficacy in the tropics. Effects of maternal immunisation on infant responses to vaccines should be explored. Prevention of infant malaria and HIV could contribute to effectiveness of immunisation programmes.

## 1 Introduction

Immunisation is key to the control of infectious diseases but the efficacy of some vaccines is poor in tropical, developing countries, where they are most needed [1]. In particular, Bacille Calmette-Guérin (BCG) immunisation has over 70% efficacy against tuberculosis in temperate countries, but low efficacy in tropical settings [2,3]. The reasons for this need to be understood. Tuberculosis remains a major cause of global morbidity and mortality [4]; effective immunisation could revolutionise tuberculosis control and new vaccines are being developed [5], but new vaccines may be influenced by the same factors that inhibit the response to BCG in the tropics. Understanding these factors may allow the development of interventions to improve the effectiveness of immunisation programmes. Several hypotheses as to the nature of these factors have been advanced. Genetic differences between populations may be important, but efficacy in migrant populations tends to approach that observed in the native populations of the adoptive country [3,6,7]; differences in BCG strains used have been considered, but trials using the same source of BCG have also shown differences in efficacy by latitude [3]; effects of vaccine exposure to sunlight and breakdown in the cold chain have been considered, but are unlikely to explain low efficacy in carefully conducted trials. Two outstanding hypotheses particularly remain to be considered.

One of these is that exposure to environmental mycobacteria, which is more common in the tropics, masks, or blocks, the response to BCG in this setting. Early evidence for this hypothesis [3] has been supported by subsequent studies showing higher levels of sensitisation to mycobacterial antigens in unvaccinated Malawian compared to British populations, and smaller increases in the gamma interferon (IFN-γ) response following BCG in Malawian than in British adolescents [8]. However, vaccine-induced responses were not directly related to prior sensitisation to environmental mycobacteria [9], suggesting that other factors might play a role. Also, differences in response to BCG immunisation were demonstrated between Malawian and British infants at an age too young for effects to be explained by direct exposure to environmental organisms [10]; thus prenatal exposures are likely to be important.

A second hypothesis is that chronic helminth infections influence responses to BCG and other vaccines [11]. Helminths elicit strong type 2 and regulatory immune responses [12]; these effects can “spill over” to influence responses to unrelated antigens and can inhibit type 1 responses that are a component of the protective response against tuberculosis [13–16]. De-worming prior to BCG immunisation can improve the induced response to purified protein derivative of *Mycobacterium tuberculosis* [17]. Also, sensitisation to helminth antigens in utero may be associated with a switch to a type 2 response profile following BCG immunisation at birth, again emphasising the potential role of exposures very early in life [18].

In response to this last observation, we set up a randomised, controlled trial of anthelminthic treatment during pregnancy to investigate the hypothesis that exposure to, and treatment of, maternal worms during pregnancy would influence the infant response to BCG and other immunisations [19]. At age one year we assessed cytokine responses induced by BCG given at birth and by tetanus immunisation given at 6, 10 and 14 weeks of age. While there is no major concern regarding efficacy of tetanus immunisation, assessment of responses to tetanus toxoid (TT) allowed us to investigate effects on a vaccine that elicits a more type-2 biased response than BCG, and that is given later in infancy. The impact of the anthelminthic intervention on cytokine responses has been reported elsewhere [20]. We here describe planned observational analyses conducted to investigate factors affecting the infant response to immunisation during pre-natal and early post-natal life.

## 2 Materials and methods

### 2.1 Study design

The study was a randomised, double-blind, placebo-controlled trial of albendazole or praziquantel treatment during pregnancy, with a 2 × 2 factorial design, resulting in fours arms, albendazole plus praziquantel, albendazole plus placebo for praziquantel, praziquantel plus placebo for albendazole and double placebo [ISRCTN32849447] [19]. Using the trial birth cohort, this observational analysis examined associations between infant cytokine responses to BCG and tetanus immunisation, and pre- and post-natal exposure to helminths, other co-infections and other potentially related factors.

### 2.2 Setting and participants

**Fig. 1:**
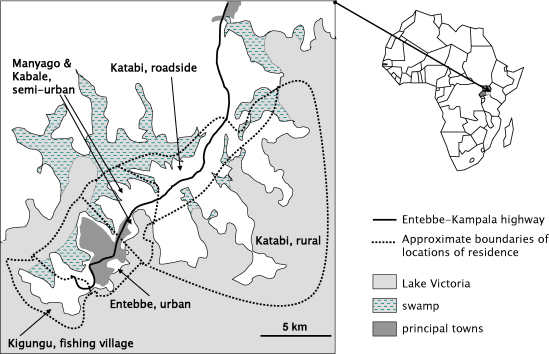
Study area.

The study area comprised Entebbe Municipality and surrounding communities (Fig. 1). Women from the study area, in the second or third trimester of pregnancy, were recruited at Entebbe Hospital antenatal clinic between 2003 and 2005 if planning to deliver in the hospital and willing to know their HIV status; they were excluded for haemoglobin <8 g/dl, clinically apparent severe liver disease, diarrhoea with blood in stool, history of adverse reaction to anthelminthics, abnormal pregnancy, or if already enrolled during an earlier pregnancy.

The study was approved by ethical committees of the Uganda Virus Research Institute and London School of Hygiene & Tropical Medicine, and by the Uganda National Council for Science and Technology. All participants gave written informed consent.

### 2.3 Procedures

Socio-demographic details were recorded and blood and stool samples obtained prior to treatment of women with the trial intervention (single dose albendazole 400 mg or matching placebo and praziquantel 40 mg/kg or matching placebo). The intervention medication was given during the second or third trimester of pregnancy (according to when the women presented at the clinic and completed screening procedures). Women received standard antenatal care including haematinics and intermittent presumptive treatment for malaria with sulfadoxine–pyrimethamine. Tetanus immunisation, up to a maximum of three doses, was given during pregnancy unless the woman had completed a total of five doses during previous pregnancies. HIV-positive women were offered single dose nevirapine for themselves and their infants for prevention of mother-to-child HIV transmission [21]. Six weeks after delivery all women received treatment with both albendazole and praziquantel. Infants received BCG and polio immunisation at birth, polio, diphtheria, pertusis, tetanus, hepatitis B and *Haemophilus influenzae* immunisation at 6, 10 and 14 weeks of age, and measles immunisation at nine months. Infant illnesses were treated at the study clinic. At age 12 months blood was obtained from infants; weight and height were measured.

Vaccines were those provided by the Ugandan National Medical Stores: during the study period, BCG vaccine was provided from three suppliers: BB-NCIPD Ltd., Bulgaria, Serum Institute of India, India and Statens Seruminstitut, Denmark.

### 2.4 Diagnostic tests, parasitology and haemotology

HIV serology was performed for mothers, and for infants aged 18 months, by rapid test algorithm [22]. HIV DNA PCR was performed [20], and HIV load measured (Bayer Versant branched DNA assay version 3.0; Bayer HealthCare, Leverkusen, Germany), for infants of HIV-positive mothers at age six weeks. Stools were examined for helminth ova by Kato-Katz method [23] and by culture for Strongyloides [24]; blood samples were examined by modified Knott's method for microfilariae [25] and by thick film for malaria parasites, as previously described [22].

Clinical malaria was defined as fever ≥37.5 °C plus parasitaemia. Asymptomatic malaria was defined as parasitaemia in the absence of fever or other symptoms of malaria.

### 2.5 Immunological assays

Primary outcomes were infant immune responses to mycobacterial antigen and to TT, taken to represent the response to BCG and tetanus immunisation, respectively. We examined stimulated cytokine production in a whole blood assay, as described elsewhere: IFN-γ was measured to assess type 1 responses; IL-5 and IL-13 were measured to assess type 2 responses (since IL-4, the hallmark of the type 2 response, is seldom detectable in culture supernatant, particularly following stimulation with mycobacterial antigen) and IL-10 was measured to assess regulatory responses [26]. Briefly, unseparated, heparinised blood was diluted to a final concentration of one-in-four using RPMI supplemented with penicillin, streptomycin and glutamine, plated in 96-well plates, and stimulated with crude culture filtrate protein from *M. tuberculosis* (cCFP; 5 μg/ml) (kindly provided by John Belisle, University of Colorado, Fort Collins, USA), TT (12 Lf/ml; Statens Seruminstitut, Denmark), phytohaemagglutinin (PHA; 10 μg/ml; Sigma, UK), or left unstimulated. Supernatants were harvested on day 6 and frozen at −80 °C until analysed.

Cytokine concentrations in supernatants were measured by ELISA (Becton Dickinson, UK). Test responses were regarded as positive if greater than the mean plus two standard deviations of negative control results for all assays: IFN-γ > 73 pg/ml; IL-5 > 34 pg/ml; IL-13 > 18 pg/ml; IL-10 > 48 pg/ml. Values below the cut-off were set to zero. Cytokine production in unstimulated test wells was subtracted from concentrations produced in response to stimulation. Assays were performed after all samples had been collected, in a randomised sequence, to avoid confounding of secular trends with variations in assay performance.

### 2.6 Study size

The study size was determined for the trial objectives, rather than for this analysis. It was anticipated that, with recruitment of 2500 women, at least 1594 infants would be assessed at one year; this would give 80% power with *p* < 0.05 to detect differences of 0.11 log_10_ in cytokine responses for exposures with two equal-sized categories [19].

### 2.7 Statistical analyses

The objective of this observational analysis was to determine socio-demographic, maternal and infant factors associated with cytokine responses following BCG and tetanus immunisation.

Socio-demographic factors were maternal age, maternal education (categories none, primary, secondary or tertiary), household socioeconomic status (a six-level score based on building materials, number of rooms, items owned) and location of residence (by zone, Fig. 1). Maternal factors were the three commonest maternal helminth infections (hookworm, *Mansonella perstans*, *Schistosoma mansoni*), maternal asymptomatic malaria parasitaemia (*Plasmodium falciparum*) and maternal immunisation status (absence or presence of a maternal BCG scar; number of documented doses of tetanus immunisation during pregnancy). Infant factors were gender, birth weight, anthropometric scores at age one year (weight-for-age, height-for-age and weight-for-height [27]), infant malaria (current, asymptomatic malaria on the day of the assay; number of documented clinical malaria episodes in the preceding year) and HIV status (based on maternal and infant serology, and infant PCR at age six weeks: unexposed, exposed-uninfected, or infected).

**Fig. 2:**
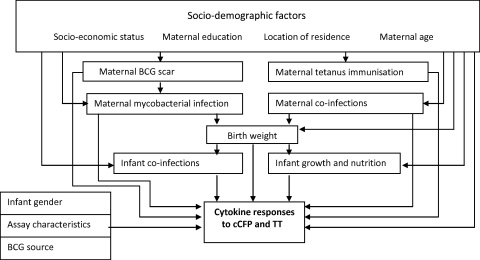
Causal diagram.

Cytokine responses showed skewed distributions, with a disproportionate number of zero values, as has commonly been observed for immunoepidemiological data and, in particular, for the use of whole blood stimulation and cytokine response assays [28–30]. Results were transformed to log_10_(cytokine concentration + 1) and analysed by linear regression using bootstrapping with 10,000 iterations to estimate standard errors and bias-corrected accelerated confidence intervals [29]. Regression coefficients and confidence limits were back-transformed to express results as ratios of geometric means. Crude associations were first examined. The following strategy was then employed to investigate multivariate associations. A simple hierarchical causal diagram was developed (Fig. 2). Socio-demographic factors were considered as potential confounders for the relationship between each exposure and cytokine response, and maternal co-infections (malaria parasitaemia and helminths) were considered as potential confounders for each other and for infant exposures. Treatment with albendazole was considered as a potential effect modifier for maternal hookworm and *M. perstans* infections, and treatment with praziquantel for *S. mansoni* infection. Infant co-infections were considered as potential confounders for infant anthropometric exposures. For all exposures, any remaining variable that showed a crude association with the outcome and that was not on the causal pathway between the exposure of interest and the outcome was included in the model to improve the precision of the estimated effects. BCG supplier (for analyses of response to BCG) and assay characteristics (antigen batch and lymphocyte count) were also considered in all models.

## 3 Results

### 3.1 Participants

**Fig. 3:**
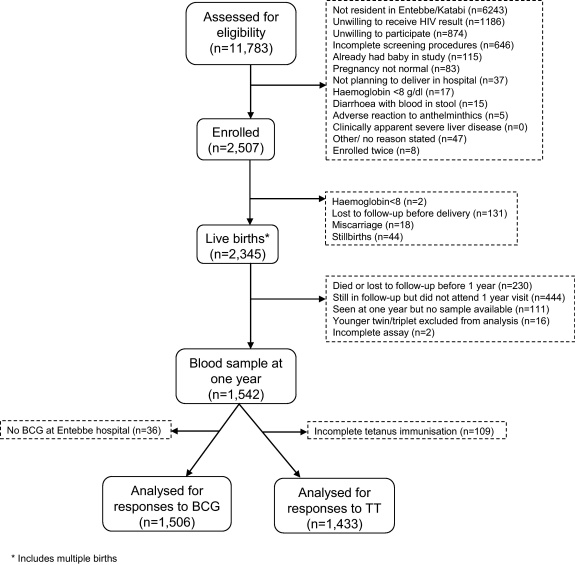
Flow of participants through the study.

The flow of participants through the study has been described elsewhere [20] and is summarised in Fig. 3. Of 2507 women enrolled, information was obtained on 2345 live births. Results from 1542 babies (singletons or older twins or triplets) were available at one year. Of these, 36 had not received BCG immunisation at Entebbe Hospital and 109 had incomplete tetanus immunisation: therefore 1506 infants were included in analyses for responses following BCG immunisation, and 1433 for tetanus immunisation.

As previously reported, the median maternal age was 23 years; most women (54%) had either primary or no formal education [31]. The majority (41%) lived in Entebbe Municipality, 28% in Manyago and Kabale, 11% Katabi roadside, 9% Katabi rural and 11% Kiggungu fishing village (Fig. 1). Sixty-eight percent had at least one helminth infection; 44% had hookworm, 21% *M. perstans* and 18% *S. mansoni*; 11% had asymptomatic malaria at enrolment; 12% had HIV infection [31]. Sixty percent had a BCG scar; 22%, 61% and 17% had zero, one and two or more recorded doses of tetanus immunisation during pregnancy, respectively. Women whose infants had cytokine results available at one year were older, of higher socioeconomic status and less likely to live in Katabi, and had lower prevalence of helminths, asymptomatic malaria and HIV infection during pregnancy, than those without results (data not shown).

Among infants with results at one year, 50% were female; the mean birth weight was 3.18 kg; at one year the mean weight-for-age *z* score was −0.33, mean height-for-age *z* score −0.84 and mean weight-for-height *z* score 0.10; 6% had asymptomatic *P. falciparum* malaria; 9% were HIV-exposed-uninfected and 1% were HIV-infected. Only 44 of 1358 infants examined had helminth infections at age one year (most common were *Ascaris* (15 infants), *Trichuris* (12 infants) and *Mansonella* (eight infants)) so effects of infant helminths were not considered in this analysis. Ninety-nine percent of infants were breast-fed to age six weeks, and 80% were still being breast-fed at age one year.

### 3.2 Cytokine responses

**Fig. 4:**
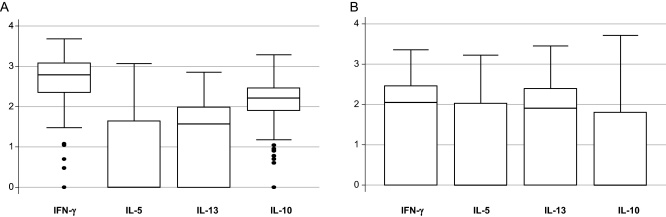
Cytokine responses measured in supernatant of a six-day whole blood assay among infants aged one year. (A) Responses to crude culture filtrate proteins of *Mycobacterium tuberculosis* among infants who received BCG immunisation at birth. (B) Responses to tetanus toxoid among infants who received three doses of tetanus immunistaion during infancy. The central line represents the median; boxes represent 75th and 25th centiles; whiskers represent upper and lower adjacent values and dots represent outside values.

Type 1 (IFN-γ) and regulatory (IL-10) cytokines were dominant in the response to cCFP; following tetanus immunisation, type 2 cytokines were more prominent (Fig. 4). Crude associations between factors examined and cytokine responses are shown in Tables 1 and 2; multivariate analyses in Tables 3 and 4.

### 3.3 Socio-demographic factors

The infant IFN-γ and IL-5 response to TT increased with maternal education, with adjusted geometric mean ratios (aGMR) (95% confidence interval (CI)) of 1.25 (1.03, 1.54) and 1.25 (1.04, 1.50) respectively, while the IL-10 response to TT was inversely associated with socio-economic status (aGMR 0.90 (0.82, 0.98)).

### 3.4 Maternal factors

Maternal *M. perstans* infection showed a positive association with infant IL-10 production in response to both cCFP (aGMR 1.23 (0.99, 1.50) and TT (aGMR 1.36 (1.04, 1.80). Both associations showed a marked interaction with maternal albendazole treatment (interaction *p*-values 0.02 and 0.001, respectively), being evident only in the albendazole-placebo group (cCFP aGMR 1.57 (1.19, 2.00) and TT aGMR 1.99 (1.35, 2.97)). No consistent associations were observed for other species.

Maternal BCG scar was associated with a markedly lower infant IL-5 and IL-13 responses to cCFP (aGMR 0.76 (0.61, 0.94) and 0.80 (0.64, 1.00)) and a somewhat lower IFN-γ response (aGMR 0.87 (0.70, 1.09)). An increasing number of doses of maternal tetanus immunisation during the pregnancy was associated with increased infant IFN-γ (aGMR 1.44 (1.16, 1.79)) and IL-13 (1.22 (1.01, 1.46)), and a weak increase in IL-5 (aGMR 1.19 (0.97, 1.44)) responses to TT.

### 3.5 Infant factors

Female infants had broadly lower responses for both cCFP and TT, with aGMRs for each cytokine response ranging from 0.69 to 0.86 (Tables 1–4).

Associations for anthropometric variables were somewhat variable; after adjustment for confounding, associations remained for the IL-13 response for TT and IL-10 response to cCFP, which both showed increased responses for higher scores: IL-13 for TT, birth weight aGMR 1.43 (1.09, 1.89), weight-for-age *z*-score at one year, 1.13 (1.01, 1.28), height-for-age *z*-score at one year 1.13 (1.01, 1.26); IL-10 for CFP, height-for-age *z*-score at one year, 1.08 (1.00, 1.17).

Current malaria parasitaemia was strongly associated with reduced IFN-γ, IL-5 and IL-13 responses for cCFP (aGMR 0.49 (0.28, 0.80), 0.41 (0.30, 0.60) and 0.46 (0.29, 0.75) respectively), and for TT (aGMR 0.47 (0.25, 0.85), 0.32 (0.21, 0.53) and 0.50 (0.26, 0.93) respectively), and with a reciprocal increase in IL-10 responses for TT (aGMR 2.38 (1.48, 3.80)). Previous episodes of malaria during infancy showed weaker effects, but a high number of episodes was associated with a reduced IL-5 response to cCFP (aGMR 0.84 (0.76, 0.95)) and an increased IL-10 response to TT (aGMR 1.18 (1.03, 1.34)).

Associations with infant HIV status differed for cCFP and TT. For cCFP, HIV-exposed-uninfected infants showed no difference in response compared to HIV-unexposed infants, but HIV-positive infants showed markedly lower IFN-γ, IL-5 and IL-13 responses (aGMR 0.06 (0.02, 0.23), 0.37 (0.25, 1.00) and 0.20 (0.09, 0.53) respectively), and higher IL-10 responses (aGMR 2.19 (1.56, 3.15)). For TT, both HIV-exposed-uninfected infants, and HIV-infected infants, showed impaired IFN-γ, IL-5 and IL-13 responses: HIV exposed-uninfected, aGMR 0.57 (0.35, 0.94), 0.51 (0.33, 0.82) and 0.61 (0.39, 0.95); HIV-infected, aGMR 0.35 (0.11, 1.13), 0.16 (0.10, 0.52) and 0.09 (0.04, 0.27); there was no effect on the IL-10 response.

## 4 Discussion

In this large birth cohort in Uganda, infant responses to BCG and tetanus immunisation were associated with pre-natal and early post-natal exposure to maternal *M. perstans* co-infection, maternal and infant HIV co-infection, and infant malaria. Maternal BCG scar showed associations with the infant response to BCG, and maternal immunisation with tetanus toxoid during pregnancy was associated with higher infant responses to their own tetanus immunisation.

As in any observational analysis, some findings may be explained by unmeasured confounders. However, most key factors identified were biological, rather than social or environmental, and adjustment for measured confounders produced little change in their effect estimates, suggesting that they are closely linked to causal mechanisms. Many statistical tests were conducted, so some apparently “significant” findings could have occurred by chance. Individual results are therefore treated with caution; rather than formally adjust for multiplicity, we focus on patterns and consistency of results, and on biological plausibility with reference to other findings.

Maternal *M. perstans* microfilaraemia was associated with enhanced IL-10 responses to both cCFP and TT in the offspring. This filarial infection is highly prevalent in Africa and central South America, but usually asymptomatic [32,33]. Adult worms inhabit serous cavities and microfilariae circulate in the blood, sometimes in thousands per millilitre, the lack of symptoms testifying to this helminth's potent immunoregulatory properties. Such helminth-induced regulation can influence host responses to unrelated antigens and IL-10 may be one key mediator of such effects [12]; among other filariases, IL-10 responses to tetanus immunisation have been found to be elevated in adults with asymptomatic *Onchocerca volvulus* infection [34,35]. Our key observation is that the non-specific effect of helminths on this regulatory cytokine response can be transmitted from mother to infant. Notably, infant IFN-γ, IL-5 and IL-13 responses were not reduced, suggesting the possibility that protective immune responses may not be impaired, and it is possible that the overall impact of exposure to maternal helminth infection in utero is an enhancement of regulatory immune responses rather than suppression of the ability to mount protective responses to vaccines and pathogens. This might be broadly beneficial, protecting against excessive inflammatory responses, including allergy [36,37].

The lack of observed effects of maternal hookworm or *S. mansoni* on type 1 and type 2 responses to mycobacterial antigens was surprising, given our own earlier findings [38], and those of Malhotra and colleagues [18]*.* However, in Malhotra's study all women had helminth infection: comparisons were made between infants sensitised and not sensitised to helminth antigens. Our study compared infants of mothers infected or not infected with each species, in a setting where most women had at least one helminth infection; moreover, for logistical reasons, a single stool sample was used for Kato Katz analysis giving limited sensitivity for diagnosis of intestinal helminths [39,40]. Both these factors may have resulted in underestimation of effects of schistosomiasis and other intestinal helminths. However, taken together with the finding (reported elsewhere [20]) that anthelminthics during pregnancy had little effect on infant responses to cCFP and TT in this study, these results suggest that maternal helminth infection may not be the major explanation for the poor efficacy of BCG immunisation in the tropics. Subsequent acquisition of helminths by the infant may be a different story [17].

Tetanus immunisation during pregnancy was associated with enhanced IFN-γ, IL-13 and (to some extent) IL-5 responses following tetanus immunisation of the offspring. These results accord with the earlier report of Gill and colleagues [41] and show that priming of the infant response to TT can be influenced by immunisation of the mother. This antigen-specific effect may result from transfer of TT across the placenta within an immune complex, utilising the immunoglobulin receptor systems involved in transfer of maternal antibody to the fetus [42–44]. Fetal exposure to antigen can result in tolerisation, but immune complexes are potent activators of the immune system, and this may explain why priming occurred in this case. The lower response to tetanus immunisation in HIV-exposed-uninfected infants may have resulted from reduced transfer of maternal antibody and antigen in this group [45,46].

By contrast, presence of a maternal BCG scar showed a negative association with infant type 2 cytokine response, and (to some extent) IFN-γ response to cCFP following BCG immunisation. This may have been a non-specific effect since maternal BCG scar was also associated with reductions in these cytokine responses to PHA (data not shown). The association was not explained by adjusting for potential confounding factors, and suggests an immunological interaction between mother and infant related to maternal mycobacterial exposure or infection. There is evidence for sensitisation to mycobacterial antigens in utero in mouse models and in humans [47,48], but tolerisation is also a possibility, and would accord with the lower response to mycobacterial antigen observed in Malawian, compared to British, infants following BCG immunisation [10]. It may be important to investigate the role of maternal mycobacterial infection, and maternal immune responses to mycobacteria, in the infant response to BCG.

Current infant malaria and infant HIV infection were associated with broad reductions in IFN-γ, IL-5 and IL-13 responses. These findings were in keeping with the recognised immunosuppressive effects of these pathogens and thus, incidentally, demonstrate the ability of this immuno-epidemiological approach to detect important effects. They contrast with the IL-10-restricted effects of maternal *M. perstans*. For malaria, these effects were mainly short-term, observed only in the presence of current infection; however, decreased IL-5 cCFP and increased IL-10 TT responses were associated with both current and previous malaria. It is possible that the independent association between increased IL-10 TT responses and household socio-economic status might be mediated by repeated, unmeasured, exposures to infection.

Consistently lower responses were seen in girls. This shows that gender differences in immune response are present at an early age, and could be related to reported gender differences in the non-specific effects of immunisation on infant mortality [49].

This study examined factors influencing the cytokine responses induced by BCG and tetanus immunisation, not their efficacy. In the case of BCG, it is likely that IFN-γ is required, although not sufficient for, protective immunity [15], while excessive production of type 2 cytokines may be detrimental [50]. Excess production of IL-10 may also be detrimental, if it is associated with suppression of protective responses, but evidence from the mouse model suggests that adequate production may be required to prevent a pathological, inflammatory response [51]. Follow up of the cohort is in progress to determine how the observed responses are related to rates of *M. tuberculosis* infection and disease. In the case of tetanus immunisation, the induction of neutralising antibody is key to protective immunity [52]; the relationship between observed effects on cytokine responses and the production of antibody will be the subject of further investigation.From a public health perspective, our results demonstrate strong effects of current, or recent infant infections on the infant response to vaccine antigens, and reinforce the importance of control and treatment of malaria and HIV infection for the immunological health of mothers and their children; but suggest that maternal helminth infection may have little, if any, adverse effect on the outcome of infant immunisation. Immunisation during pregnancy may enhance the infant response to selected vaccines, and this, as well as the role of prior maternal BCG immunisation and mycobacterial infection in determining the infant response to BCG immunisation, needs to be explored in further research.

## Figures and Tables

**Table 1:** Crude associations with response to crude culture filtrate proteins of *Mycobacterium tuberculosis* in infants who received BCG immunisation at birth.

Factor^a^	IFN-γ	IL-5	IL-13	IL-10
Socio-demographic characteristics				
Household socioeconomic status	1.02 (0.94, 1.11)	1.05 (0.97, 1.15)	1.04 (0.95, 1.14)	0.99 (0.91, 1.07)
Mother's education	1.03 (0.88, 1.19)	0.99 (0.86, 1.15)	1.03 (0.88, 1.21)	0.92 (0.80, 1.05)
Mother's age at enrolment	1.01 (0.99, 1.03)	**1.02 (1.00, 1.03)**	1.01 (0.99, 1.03)	1.00 (0.98, 1.02)
Location of residence				
Entebbe Town, urban	1	1	1	1
Manyago and Kabale, semi-urban	1.07 (0.82, 1.37)	1.01 (0.79, 1.29)	0.93 (0.72, 1.21)	1.01 (0.79, 1.27)
Kigungu, fishing village	1.10 (0.76, 1.51)	1.00 (0.71, 1.44)	0.98 (0.68, 1.39)	1.19 (0.85, 1.60)
Katabi, roadside	1.15 (0.76, 1.66)	0.87 (0.63, 1.27)	1.34 (0.92, 1.92)	1.36 (0.96, 1.83)
Katabi, rural	0.91 (0.54, 1.40)	**0.66 (0.46, 0.99)**	0.86 (0.54, 1.35)	**1.67 (1.12, 2.30)**
Maternal characteristics				
Maternal hookworm infection at enrolment	0.89 (0.71, 1.11)^b^	0.93 (0.76, 1.15)	1.01 (0.81, 1.26)	0.91 (0.75, 1.10)
Maternal *S. mansoni* infection at enrolment	0.80 (0.58, 1.07)	0.83 (0.64, 1.08)	0.91 (0.68, 1.20)	**0.71 (0.53, 0.93)**
Maternal *M. perstans* infection at enrolment	0.95 (0.72, 1.21)	0.82 (0.64, 1.04)	0.95 (0.72, 1.23)	**1.31 (1.03, 1.63)**^c^
Maternal malaria parasitaemia at enrolment	0.88 (0.57, 1.23)	**0.61 (0.46, 0.86)**	0.81 (0.56, 1.16)	1.33 (0.95, 1.71)
Maternal BCG scar	0.85 (0.68, 1.06)	**0.77 (0.62, 0.95)**	**0.77 (0.62, 0.97)**	1.00 (0.82, 1.22)
Infant characteristics				
Infant female	**0.77 (0.63, 0.95)**	**0.74 (0.61, 0.91)**	**0.79 (0.64, 0.98)**	0.85 (0.70, 1.03)
Birth weight	1.21 (0.97, 1.53)	1.17 (0.93, 1.48)	1.27 (0.98, 1.63)	1.14 (0.93, 1.42)
Weight for age *z* score at 1 year	**1.10 (1.01, 1.21)**	**1.09 (1.00, 1.19)**	1.04 (0.95, 1.14)	**1.09 (1.01, 1.18)**
Height for age *z* score at 1 year	1.06 (0.97, 1.16)	**1.10 (1.01, 1.20)**	1.08 (0.99, 1.18)	1.05 (0.97, 1.14)
Weight for height *z* score at 1 year	1.09 (0.99, 1.19)	1.05 (0.97, 1.14)	1.00 (0.91, 1.09)	1.07 (0.99, 1.16)
Infant malaria (current, asymptomatic)	**0.47 (0.27, 0.75)**	**0.43 (0.32, 0.62)**	**0.48 (0.30, 0.77)**	0.97 (0.63, 1.35)
Number of malaria episodes in infancy	0.97 (0.85, 1.09)	**0.84 (0.76, 0.94)**	1.00 (0.88, 1.12)	1.09 (0.96, 1.21)
Infant HIV status				
Unexposed	1	1	1	1
Exposed, uninfected	1.00 (0.64, 1.44)	1.00 (0.72, 1.44)	1.12 (0.77, 1.63)	0.87 (0.59, 1.21)
Infected	**0.06 (0.02, 0.22)**	**0.33 (0.26, 0.74)**	**0.16 (0.09, 0.38)**	**2.20 (1.49, 3.21)**

Data are for 1506 children who received BCG immunisation at Entebbe Hospital. Confidence intervals not including one are highlighted in bold.

a Missing values: maternal BCG scar, 54; maternal malaria, 26; maternal education, 3; household socioeconomic status, 32; location of residence, 15; birth weight 248; weight for age, 2; height for age, 16; weight for height *z* scores, 17; infant malaria parasitaemia, 28.

b Effect of maternal hookworm on IFN-γ response modified by maternal treatment with albendazole: albendazole group, GMR = 0.67 (0.48, 0.90); placebo group GMR = 1.19 (0.88, 1.61).

c Effect of maternal *M. perstans* on IL-10 response modified by maternal treatment with albendazole: albendazole group, GMR = 0.97 (0.66, 1.36); placebo group GMR = 1.69 (1.27, 2.19).

**Table 2:** Crude associations for infant response to tetanus toxoid after tetanus immunisation at 6, 10 and 14 weeks of age.

Factor^a^	IFN-γ^b^	IL-5	IL-13	IL-10
Socio-demographic characteristics				
Household socioeconomic status	1.09 (0.97, 1.23)	1.03 (0.92, 1.15)	1.03 (0.93, 1.14)	**0.91 (0.82, 1.00)**
Mother's education	**1.22 (1.00, 1.49)**	**1.22 (1.01, 1.47)**	1.15 (0.97, 1.36)	0.88 (0.75, 1.03)
Mother's age at enrolment	0.98 (0.96, 1.01)	1.00 (0.98, 1.03)	0.99 (0.97, 1.01)	0.99 (0.97, 1.01)
Location of residence				
Entebbe Town, urban	1	1	1	1
Manyago and Kabale, semi-urban	0.88 (0.62, 1.22)	0.82 (0.61, 1.13)	0.77 (0.58, 1.04)	0.94 (0.72, 1.23)
Kigungu, fishing village	1.00 (0.61, 1.64)	1.12 (0.72, 1.75)	1.02 (0.67, 1.50)	1.31 (0.90, 1.94)
Katabi, roadside	1.13 (0.67, 1.83)	1.34 (0.84, 2.11)	1.33 (0.86, 1.94)	**1.48 (1.01, 2.18)**
Katabi, rural	0.92 (0.50, 1.66)	0.61 (0.36, 1.08)	0.65 (0.37, 1.09)	**2.00 (1.23, 3.28)**
Maternal characteristics				
Maternal hookworm infection at enrolment	0.85 (0.65, 1.14)	0.95 (0.73, 1.22)^c^	0.87 (0.69, 1.10)^d^	1.17 (0.94, 1.46)
Maternal *S. mansoni* infection at enrolment	0.88 (0.61, 1.29)	0.97 (0.69, 1.36)	0.96 (0.69, 1.30)	0.81 (0.61, 1.08)
Maternal *M. perstans* infection at enrolment	0.87 (0.61, 1.24)	1.03 (0.75, 1.44)	0.95 (0.70, 1.27)	**1.46 (1.11, 1.95)**^e^
Maternal malaria parasitaemia at enrolment	0.85 (0.52, 1.38)	0.70 (0.45, 1.09)	0.79 (0.51, 1.20)	1.09 (0.74, 1.61)
Number of doses of tetanus toxoid during pregnancy	**1.45 (1.18, 1.79)**	1.21 (0.99, 1.47)	**1.25 (1.05, 1.50)**	0.86 (0.72, 1.02)
Infant characteristics				
Infant female	**0.70 (0.53, 0.93)**	0.78 (0.61, 1.01)	0.85 (0.67, 1.08)	0.77 (0.62, 0.95)
Birth weight	1.06 (0.77, 1.47)	1.28 (0.95, 1.69)	**1.43 (1.08, 1.87)**	0.88 (0.69, 1.13)
Weight for age *z* score at 1 year	1.11 (0.98, 1.25)	1.19 (1.07, 1.32)	**1.20 (1.09, 1.33)**	1.04 (0.94, 1.15)
Height for age *z* score at 1 year	1.10 (0.98, 1.23)	1.14 (1.02, 1.27)	**1.22 (1.11, 1.35)**	**0.89 (0.81, 0.98)**
Weight for height *z* score at 1 year	1.08 (0.96, 1.22)	1.15 (1.03, 1.29)	**1.12 (1.01, 1.24)**	**1.14 (1.03, 1.25)**
Infant malaria (current, asymptomatic)	**0.45 (0.24, 0.83)**	0.32 (0.20, 0.54)	**0.45 (0.26, 0.74)**	**2.73 (1.65, 4.43)**
Number of malaria episodes in infancy	0.92 (0.78, 1.08)	0.90 (0.79, 1.05)	**0.87 (0.75, 0.98)**	**1.24 (1.09, 1.41)**
Infant HIV status				
Unexposed	1	1	1	1
Exposed, uninfected	**0.57 (0.34, 0.94)**	**0.53 (0.35, 0.84)**	0.67 (0.43, 1.02)	0.94 (0.64, 1.41)
Infected	**0.30 (0.10, 1.00)**	**0.15 (0.09, 0.59)**	**0.09 (0.04, 0.25)**	0.81 (0.34, 2.64)

Data are for 1433 children who received three doses of tetanus immunisation during infancy. Confidence intervals not including one are highlighted in bold.

a Missing values: maternal doses of tetanus immunisation, 2; maternal malaria, 24; maternal education, 3; household socioeconomic status, 28; location of residence, 15; birth weight 233; height and weight for height *z* scores, 12; infant malaria parasitaemia, 26.

b One additional missing value for the IFN-γ response to tetanus toxoid.

c Effect of maternal hookworm on IL-5 response modified by maternal treatment with albendazole: albendazole group, GMR = 0.70 (0.48, 1.01); placebo group GMR = 1.26 (0.87, 1.82).

d Effect of maternal hookworm on IL-13 response modified by maternal treatment with albendazole: albendazole group, GMR = 0.61 (0.44, 0.86); placebo group GMR = 1.23 (0.88, 1.75).

e Effect of maternal *M. perstans* on IL-10 response modified by maternal treatment with albendazole: albendazole group, GMR = 0.95 (0.65, 1.43); placebo group GMR = 2.17 (1.46, 3.21).

**Table 3:** Factors associated with the cytokine response to crude culture filtrate proteins of *Mycobacterium tuberculosis* in infants who received BCG immunisation at birth, after adjustment for potential confounders and other crudely associated factors.

Cytokine/factor	Adjusted GMR (95% CI)^a^
Interferon-γ	
Maternal hookworm infection	
Albendazole treatment group	0.70 (0.50, 0.96)
Albendazole placebo group	1.29 (0.95, 1.76)
Baby female	0.79 (0.64,0.98)
Baby malaria (current, asymptomatic)	0.49 (0.28,0.80)
Baby HIV status	
Unexposed	1
Exposed, uninfected	0.96 (0.62,1.36)
Infected	0.06 (0.02,0.23)
Interleukin-5	
Maternal BCG scar	0.76 (0.61, 0.94)
Baby female	0.72 (0.58, 0.89)
Baby malaria (current, asymptomatic)	0.41 (0.30, 0.60)
Number of malaria episodes during infancy	0.84 (0.76, 0.95)
Baby HIV status	
Unexposed	1
Exposed, uninfected	1.01 (0.71, 1.49)
Infected	0.37 (0.25, 1.00)
Interleukin-13	
Maternal BCG scar	0.80 (0.64, 1.00)
Baby malaria (current, asymptomatic)	0.46 (0.29, 0.75)
Baby HIV status	
Unexposed	1
Exposed, uninfected	1.09 (0.74, 1.59)
Infected	0.20 (0.09, 0.53)
Interleukin-10	
Maternal *S. mansoni*	0.76 (0.58, 0.97)
Maternal *M. perstans*	
Albendazole treatment	0.93 (0.66, 1.27)
Albendazole placebo	1.57 (1.19, 2.00)
Baby height-for-age *z*-score at 1 year	1.08 (1.00, 1.17)
Baby HIV status	
Unexposed	1
Exposed, uninfected	0.83 (0.58, 1.14)
Infected	2.19 (1.56, 3.15)

a Figures are geometric mean ratios of cytokine production, with bootstrap 95% confidence intervals.

**Table 4:** Factors remaining associated with the cytokine response to tetanus toxoid in infants who received tetanus immunisation at 6, 10 and 14 weeks of age, after adjustment for potential confounders and other crudely associated factors.

Cytokine/factor	Adjusted GMR (95% CI)^a^
Interferon-γ	
Maternal education	1.25 (1.03, 1.54)
Number of doses of tetanus toxoid during pregnancy	1.44 (1.16, 1.79)
Baby female	0.69 (0.52, 0.91)
Baby malaria (current, asymptomatic)	0.47 (0.25, 0.85)
Baby HIV status	
Unexposed	1
Exposed, uninfected	0.57 (0.35, 0.94)
Infected	0.35 (0.11, 1.13)
Interleukin-5	
Maternal education	1.25 (1.04, 1.50)
Baby malaria (current, asymptomatic)	0.32 (0.21, 0.53)
Baby HIV status	
Unexposed	1
Exposed, uninfected	0.51 (0.33, 0.82)
Infected	0.16 (0.10, 0.52)
Interleukin-13	
Location of residence	
Entebbe Town	1
Manyago and Kabale	0.99 (0.64, 1.47)
Kigungu	0.75 (0.56, 0.99)
Katabi, roadside	1.30 (0.86, 1.92)
Katabi, rural	0.60 (0.35, 1.02)
Maternal hookworm	
Albendazole treatment	0.64 (0.45, 0.88)
Albendazole placebo	1.33 (0.95,1.89)
Number of doses of tetanus toxoid during pregnancy	1.22 (1.01, 1.46)
Birth weight	1.43 (1.09, 1.89)
Weight for age *z* score 1 year	1.13 (1.01, 1.28)
Weight for height *z* score 1 year	1.13 (1.01, 1.26)
Baby malaria (current, asymptomatic)	0.50 (0.26, 0.93)
Baby HIV status	
Unexposed	1
Exposed, uninfected	0.61 (0.39, 0.95)
Infected	0.09 (0.04, 0.27)
Interleukin-10^2^	
Household socioeconomic status	0.90 (0.82, 0.98)
Maternal *M. perstans*	
Albendazole treatment	0.87 (0.60, 1.30)
Albendazole placebo	1.99 (1.35, 2.97)
Baby female	0.78 (0.63, 0.97)
Baby malaria (current, asymptomatic)	2.38 (1.48, 3.80)
Total malaria episodes in infancy	1.18 (1.03, 1.34)

a Figures are geometric mean ratios of cytokine production, with bootstrap 95% confidence intervals.
